# Quality of Life Variables Assessment, Before and After Pancreatoduodenectomy (PD): Prospective Study

**DOI:** 10.5539/gjhs.v8n6p203

**Published:** 2015-11-17

**Authors:** Μaria Arvaniti, Νikolaos Danias, Εleni Theodosopoulou, Vassilis Smyrniotis, Μ. Karaoglou, Pavlos Sarafis

**Affiliations:** 1 “Attikon” University General Hospital (Chaidari), Athens, Greece; 2 National and Kapodistrian University of Athens, Nursing Department, Athens, Greece; 3 Cyprus University of Technology, Nursing Department, Limassol, Cyprus

**Keywords:** quality of life, pancreatic cancer, preoperative and postoperative care of patients with pancreatoduodenectomy (PD)

## Abstract

**Introduction:**

The treatment of pancreatic cancer is a complex problem, due to late diagnosis, the need for specialized surgical treatment, the large number of relapses and poor survival.

**Objective:**

To evaluate the quality of life of patients with periampulary pancreatic cancer before and after pancreatoduodenectomy (PD).

**Material and Method:**

The sample was collected in the “Attikon” University General Hospital (Chaidari). It consists of 20 subjects with a mean age of 65.9 years (SD = 10,2 years). For the quality of life measurement, we used the (EORTC) QLQ-C30 version 3.0., as well as the EORTC QOL-PAN26.

**Results:**

From the sample of 20 patients who participated, full data were collected for 18 of them during the first month, 17 during the third month and 16 during the sixth month. Regarding symptoms, as they were recorded with the QLQ-30 questionnaire, there was a significant increase of fatigue, a significant reduction of pain and constipation, while economic difficulties increased. As for the mean and median values for the dimensions of the PAN-26 questionnaire during monitoring, there was a significant decrease in pancreatic and liver pain symptoms during follow-up, while the gastrointestinal symptoms increased in frequency. In addition, the body image and sexuality worsened.

**Conclusions:**

The surgical treatment of pancreatic cancer with pancreatoduodenectomy (PD), according to the early survey data using the (EORTC) QLQ-C30 version3.0, and the EORTC QOL-PAN26 questionnaires, seems to have a favorable impact on quality of life, as evidenced by the improvement of most parameters evaluated during the study period.

## 1. Introduction

Pancreatic neoplastic disease is a relatively uncommon nosologic entity in clinical practice. Benign tumors create cystic formations called cystadenomas. Associated progression to malignancy has not been documented. Nevertheless, malignant tumors are mainly adenocarcinomas involving the ductal system (Avgoustis, Papageorgiou, Kintzonidis, Olympitis, & Papaioannou, 1982). The remaining of the lesions arises from the cells of the pancreatic islets.

International epidemiological studies suggest that over 200,000 people die annually from pancreatic cancer (PC). It is the fourth cause of death from cancer worldwide with the highest incidence and death rates observed in developed countries. In Greece, the expected PC prevalence in the general population appears remarkably low (3:100,000) compared to the reported incidence globally (8:100.000) (Freelove & Walling, 2006). Like many other types of cancer, PC is a disease of older people, with an average age of first diagnosis at 71 years. It is also common in smokers. Men as well as Afro-Americans are disproportionately more affected. Relevant important risk factors are chronic pancreatitis due to long-term alcohol abuse, obesity, and diabetes mellitus (National Cancer Institute, 2014).

The treatment of PC remains problematic, due to late diagnosis, the need for specialized surgical approach, inadequate excision and limited survival. Surgical resection however, remains the therapeutic intervention of choice (Krespis, Stamou, & Bantias, 2009). PC is operable in less than 20% of patients where involvement of iliac and mesenteric plexus is excluded. Minimal involvement of the mesenteric and portal vein or hepatic artery remains controversial. In case of location on the pancreatic head, the procedure of choice is pancreatoduodenectomy (PD) with regard to removal of the head of the pancreas along with the duodenum and the proximal jejunum, the gall bladder and common bile duct and distal stomach. The pancreatic, upper mesenteric and hepato-duodectum lymph nodes are excised. A modified Whipple procedure may also be applied with referral to pylorus preservation.

Neoplastic disorders may affect seriously everyday life of the patient, his/her family life, emotional and economic situation and many other aspects of personal life. This effect depends on several factors such as: a) parameters independent of disease severity and quality of health services, but relevant to the patient’s personality (age, sex, socioeconomic status, attitude towards the disease and life), b) factors related directly to the disease (disease severity, frequency of relapses, necessity of hospitalization) and finally c) therapy applied, pharmacy, surgery and its effect on the patient’s life and quality of care (Bottomley, Vanvoorden, Flechtner & Therasse, 2003).

Quality of life measurement in patients with PC takes often place during the last 20 years; the same phenomenon is also bibliographically observed, with other chronic and neoplastic diseases. During the period 1980-1996, the number of citations that refer to the quality of life of patients with PC has increased by 25 times. However, in fewer than 20% of the studies the quality of life of PC patients was assessed with a validated questionnaire (Billings, Cbristein, Harmsen, Harrington, & Chari, 2005).

The purpose of the proposed research is to fill the knowledge gap resulting from the study of the clinical symptoms that occur from the PD as well as to provide information on the quality of life of patients with periampulary pancreatic tumor(s) before and after the surgical procedure.

## 2. Material and Methods

The sample consists of 20 individuals with an average age of 65.9 years (SD = 10,2 years). Among study participants, complete data during the first month were collected from 18 patients, during the third month from 17 and during the sixth month from 16 patients.

The tools used for data collection were the (EORTC) QLQ-C30 and the (EORTC) QOL-PAN26 questionnaires. The QLQ C-30 EORTC comprises of one question on general health (global health -GH), the exploration of five functional areas such as physical functioning-PF, role functioning-RF, emotional functioning -EF, cognitive functioning-CF, and social functioning-SF, as well as scales regarding 8 symptoms: fatigue, pain, nausea-vomiting, shortness of breath, loss of appetite, sleep disturbances, constipation, diarrhea, and 1 element regarding the economic impact of the disease (financial impact -FI). The scoring of the EORTC QLQ C-30 at all scales was ranging from 0 to 100 (Kontodimopoulos, Samartzis, Papadopoulos & Niakas, 2012). The EORTC QOL-PAN26 includes 26 questions relating to disease symptoms, side effects of treatment and emotional consequences especially for pancreatic cancer (Fitzsimmons et al., 1995).

The increase of values in the QLQ-30 questionnaire, regarding the “Symptoms” category, equals to worse results. The increase of values in the QLQ-30 questionnaire, regarding the “Functionality” category, equals to better results. The increase of values in all categories equals to worse results, with the exception of the “Satisfaction with health services” and the “Sexuality” categories, where the increase equals to better results.

To describe the quantitative variables, we used mean values, standard deviations (Standard Deviation = SD) and medians. For the description of qualitative variables, we used absolute (N) and relative (%) frequencies. Some mixed linear regression models were used to inspect the dimensional variations of the QLQ-30 and PAN-26 questionnaires during monitoring. The analysis was done using logarithmic transformations due to the asymmetry of the distributions. Significance levels are bilateral and statistical significance was set at 0.05. For the analysis we used the statistical program STATA 11.0.

## 3. Results

Table 1 shows the demographic characteristics of patients participating in the study. Half of the patients (50%) were male and the majority of participants (65%) were married. Furthermore, 65% were retired and the majority of patients (90%) lived with a spouse or their children. 35% were smokers and 10% former smokers.

**Table 1:** Demographics of the sample

	N (%)
**Gender**	
Women	10 (50,0)
Men	10 (50,0)
Age, mean value (SD)	65,9 (10,2)
**Education**	
None	1 (5,0)
Primary School	7 (35,0)
Junior High School	3 (15,0)
High School/Lyceum	3 (15,0)
Higher Education	3 (15,0)
Master	3 (15,0)
**Family status**	
Single	3 (15,0)
Married	13 (65,0)
Divorced - Separated	4 (20,0)
Children	
No	1 (5,0)
Yes	19 (95,0)
Number of children, mean value (SD)	2 (0,9)
**Occupation/Work**	
Pensioner	13 (65,0)
Full-time job	3 (15,0)
Housewife	4 (20,0)
**Living Situation**	
Alone	1 (5,0)
With the spouse and/or the children	18 (90,0)
With a caregiver	1 (5,0)
Other	2 (10,0)
**Smoking**	
No	11 (55,0)
Yes	7 (35,0)
Ex-smoker	2 (10,0)

The clinical characteristics of the patients are presented in Table 2. 85% suffered from concomitant physical illness and 15.8% presented with some sort of mental illness. The majority of patients (75%) were under medical treatment. The cause for initial hospitalization was mainly cancer spotted on the head of the pancreas; there were three cases of cancer of the duodenum, and one patient with neoplastic lesion of the lower end of bile duct. Jaundice was the predominant symptom (55%), followed by diarrhea (30%) and pain (30%). The mean duration of the disease was 3.2 months (SD = 2.7 months), and in 15% of the patients a biliary duct stent had been placed. Positive lymph nodes were present in 90% of the cases, while in 57.9% of them there were metastases.

**Table 2:** Clinical characteristics of the patients

	Ν (%)
Concomitant physical disease	17 (85,0)
Chronic mental illness	3 (15,8)
Systemic medication taking	15 (75,0)
**Cause**	
Cancer at the top of the pancreas	17 (85,0)
Duodenum cancer	3 (15,0)
Cancer of the lower part of the gall Chronic pancreatitis	1 (5,0) 1(5,0)
**Symptoms**	
Jaundice	11 (55,0)
Indigestion	3 (15,8)
Diarrhea	6 (30,0)
Pain	6 (30,0)
**Tumor size, mean value (SD)**	**8 (3,8)**
**Existence of lymph nodes**	18 (90,0)
**Existence of metastasis**	11 (57,9)
**Previous treatments**	
No	17 (85,0)
ERCP –Stent placement	3 (15,0)
Duration of current disease (months):	3,2 (2,7)
**Weight change**	19 (95,0)

Complications occurred in 4 cases during early postoperative period. In two cases there was an anastomotic leak, while in the other two cases respiratory failure occurred. The average length of hospital stay was 11.8 days (SD = 8.3 days) and there was no perioperative death. One month later, there was no death; there was re-hospitalization in 50% of the cases, and postoperative complications in 66.7%. Postoperative complications were related to anastomotic leak in 3 cases, to fluid collection in another 3 cases, to respiratory failure in 2 cases, and to acute pancreatitis in 1 case. In three months, one patient died; there was re-hospitalization in 20% of the cases, while there were no postoperative complications. In six months there were no more deaths or complications related to surgery. One patient had to be re-hospitalized, because he/she needed transfusion due to CHMTH (Chemotherapy).

The mean and median values of the dimensions of the QLQ-30 questionnaire during monitoring are presented in Table 3. There was no significant change in the general health indicator during monitoring. Also, the functionality dimensions have not changed much, except for the social functioning dimension, where there was a reduction of levels during the six months. Regarding symptoms as recorded in the QLQ-30 questionnaire, a significant increase of fatigue was found, as well as significant reduction of pain and constipation, while economic difficulties increased.

**Table 3:** Changes in the dimensions of the QLQ-30 questionnaire during monitoring

	Originally		1 month		3 months		6 months		
	Mean Value (SD)	Median	Mean Value (SD)	Median	Mean Value (SD)	Median	Mean Value (SD)	Median	P
***Global health status Functionality***	62,5 (15,5)	66,7	54,2 (16,4)	58,3	54,2 (9,6)	50,0	56,3 (13,4)	50,0	0,467
Physical functionality	82,9 (23)	90,0	62,9 (28,4)	73,3	62,1 (14,3)	60,0	62,7 (18,5)	60,0	0,858
Part in life	78,1 (28,4)	91,7	60,4 (28,5)	66,7	56,3 (25)	66,7	53,1 (26,7)	50,0	0,590
Emotional functionality	84,9 (20,2)	91,7	88,5 (15,5)	100,0	89,6 (18,6)	100,0	88 (15,8)	91,7	0,442
Cognitive functionality	95,8 (16,7)	100,0	96,9 (9,1)	100,0	95,8 (12,9)	100,0	100 (0)	100,0	0,185
Social functionality	92,7 (18,2)	100,0	67,7 (29,5)	66,7	50 (21,1)	66,7	53,1 (18,5)	66,7	0,047
***Symptoms***									
Fatigue	24,3 (25,4)	16,7	30,6 (23,5)	27,8	31,9 (24)	27,8	35,4 (20,4)	27,8	0,029
Nausea and vomit	3,1 (6,7)	0,0	12,5 (28,9)	0,0	6,3 (17,1)	0,0	4,2 (16,7)	0,0	0,214
Pain	25 (18,3)	25,0	14,6 (22,7)	8,3	3,1 (6,7)	0,0	10,4 (18,1)	0,0	0,006
Breathlessness	2,1 (8,3)	0,0	4,2 (11,4)	0,0	0 (0)	0,0	0 (0)	0,0	0,200
Insomnia	6,3 (13,4)	0,0	10,4 (20,1)	0,0	6,3 (18,1)	0,0	10,4 (20,1)	0,0	0,939
Loss of appetite	12,5 (20,6)	0,0	22,9 (26,4)	16,7	27,1 (30,4)	33,3	27,1 (30,4)	16,7	0,331
Constipation	18,8 (24,2)	10,0	6,3 (13,4)	0,0	2,1 (8,3)	0,0	0 (0)	0,0	0,001
Diarrhea	18,8 (36,5)	0,0	29,2 (26,9)	33,3	35,4 (22,7)	33,3	29,2 (26,9)	33,3	0,112
Financial Difficulties	6,7 (18,7)	0,0	47,9 (54,4)	33,3	27,1 (18,1)	33,3	31,3 (19,1)	33,3	<0,001

Finally, Table 4 shows the mean and median values for the dimensions of the PAN-26 questionnaire during monitoring. We found a significant reduction of pancreatic and liver pain symptoms during monitoring, while the digestive symptoms increased in frequency. Additionally, body image and sexuality worsened.

**Table 4:** Changes in the dimensions of the PAN-26 questionnaire during monitoring

	Originally		1 month		3 months		6 months		
	Mean Value (SD)	Διμεσος	Mean Value (SD)	Διμεσος	Mean Value (SD)	Διμεσος	Mean Value (SD)	Διμεσος	P
Pancreatic pain	15,1 (11,1)	8,3	11,5 (13,6)	8,3	1,6 (4,5)	0,0	1,1 (4,3)	0,0	<0,001
Digestive symptoms	29,2 (23,2)	33,3	40 (23,4)	33,3	47,9 (19,1)	50,0	47,9 (16)	41,7	0,017
Altered bowel movement	19,8 (32,9)	0,0	5,2 (10)	0,0	9,4 (12,1)	0,0	7,3 (14,9)	0,0	0,467
Hepatic	25 (30,4)	10,0	1,1 (4,3)	0,0	0,0 (0,0)	0,0	2,2 (8,6)	0,0	0,002
Body image	14,6 (20,1)	8,3	31,3 (29,1)	25,0	41,7 (26,5)	33,3	45,8 (18,8)	41,7	<0,001
Satisfaction with health care	63,5 (10,9)	66,7	67,7 (9,6)	66,7	68,8 (10,3)	66,7	64,6 (14,8)	66,7	0,905
Sexuality	50 (29,8)	50,0	28,9 (17,2)	33,3	11,1 (16,3)	0,0	14,6 (17,1)	0,0	<0,001

## 4. Discussion

International literature on surgery of PC and especially PD is abundant. Relating to the effects on quality of life before and after PD, data are more limited. Although the research tools, (EORTC) QLQ-C30 and the (EORTC) QOL-PAN26 questionnaires, used to measure the quality of life of people with malignant diseases, are very widespread in general, surveys regarding pancreatic cancer are limited.

Unfortunately, pancreatic cancer is a disease that has no specific symptoms and is commonly diagnosed when it has already spread to other organs. The most common symptoms include pain mainly epigastric, unexplained weight loss, jaundice, discolored stool and nausea (Wolfgang et al., 2013). In this study, jaundice was the predominant symptom, followed by diarrhea and pain. Huang et al. (2000) in their study with a sample of 323 concerning the quality of life assessment, as well as the functional outcome of patients after PD, expressed that jaundice and pain are the main symptoms. Many patients also report weight loss, steatorrhea, and diabetes that develop in 20-50% of patients, who eventually develop exocrine and endocrine failure (McClaine et al., 2009; Tran, vam Lanschot, Bruno, & van Eijck, 2010).

Kostro and Śledziński (2008) in their study intended to evaluate changes in quality of life (QoL) of patients using the (EORTC) QLQ-C30 and the (EORTC) QOL-PAN26 questionnaires, after surgery for PC. The study included a total of 54 patients (26 of which had undergone a PD). Regarding the symptoms, as recorded by a QLQ-30 questionnaire, significant improvement was observed in the evaluation of pain, fatigue and dyspnea whereas the symptom of diarrhea was not improved. In our study, as far as the symptoms recorded in the QLQ-30 questionnaire are concerned, there was a substantial increase in fatigue, while a significant reduction of pain and constipation was also recorded. In other studies, too, PD was found to result in improved pain control and as well as quality of life in general (Russell & Theis, 2003).

According to the dimensions of the PAN-26 questionnaire during monitoring, a significant reduction of pancreatic and liver pain symptoms was found, as was the case in other studies, (Kostro & Śledziński, 2008; McClaine et al., 2009) and the digestive symptoms increased.

Conversely, Patti et al. (1987) and the Fink et al. (1988) when evaluating the gastrointestinal function of patients after PD, they found that many patients had mild gastrointestinal symptoms. And McLeod et al. (1995) emphasize that the quality of life assessment and the nutrition of patients after PD was good.

It is obvious that patients undergoing surgical resection of the pancreas may experience some postoperative complications that may influence their lifestyle (Pezzilli, 2006). Risks that may arise from the process of surgical excision are related mainly to intraoperative time, estimated blood loss, duration of hospital stay (Russell & Theis, 2003), as well as other major complications (Huang et al., 2000).

Although mortality from pancreatic resection has been reduced over the years, it is still associated with significant morbidity that occurs in 40-60% of the patients (Tran et al., 2010). Complications related to pancreatic excision include pancreatic fistula, (Huang et al., 2000) delayed gastric emptying in up to 40% of patients (Tran et al., 2010; Saraee, Vahedian-Ardakani, Saraee, Pakzad, & Wadji, 2015), intraabdominal abscess (Saraee et al., 2015), anastomotic leak (McClaine et al., 2009) surgical site infection, pneumonia (Sukharamwala, Thoens, Szuchmacher, Smith, & DeVito, 2012; Saraee et al., 2015), intraabdominal hemorrhage, bleeding from the gastrointestinal tract, and other minor complications (Pezzilli et al., 2011). In our study, postoperative complications were mainly related to anastomotic leak, fluid collection, respiratory failure, and acute pancreatitis.

During the follow-up period, however, a significant effect is observed, as time passed by, on a physical and emotional level since the physical capacity and the feelings are improved, according to the study, after the ectomy (Kostro & Śledziński, 2008). On the contrary, in the study of Huang et al. (2000), patients who underwent pancreatectomy for chronic pancreatitis and pancreatic adenocarcinoma had significantly lower QOL scores in the areas of physical and psychological function, compared to patients with laparoscopic cholecystectomy, as well as healthy individuals. As far as cognitive and social functioning were concerned, there was no great improvement. In our study, there was no significant change in the functionality dimensions, except for the social functionality dimension, where there was a reduction of the levels during the six months. However, according to the study of Andersson et al. (2012) patients after PD managed to recover “former selves” and their social life. Social factors include support from family, friends and acquaintances, as well as the financial status of the patient (Kostro and Śledziński, 2008). In this study the economic difficulties increased. Moreover, the body image and sexuality got worse.

Chan et al. (2012) in their study, with a sample of 37 patients who underwent PD, observed for a time period of two years, the progress in quality of life. The SF-36 questionnaire that generally counts health-related items was distributed, pre-operatively, and at one month, six months and twelve months postoperatively. Initially, in the first month, physical and emotional functioning was reduced while at 12 months there was a significant improvement. The researchers concluded thatPD has a positive effect on quality of life, as evidenced by the improvement of most parameters evaluated in the postoperative period.

There was no significant change in the general health indicator during monitoring, according to the values of the scale of the QLQ-30 questionnaire. And in the study of McClaine et al. (2009), patients (Whipple PD - 59 patients) showed no significant differences in the evaluation of their overall health. The quality of life was measured by the EORTC QLQ questionnaire. Morbidity and mortality was improved after one month, while postoperative pain and quality of life of the patients were improved at 24 months and at 63 months.

The average length of hospital stay in this study was 11.8 days (SD = 8.3 days), time comparable, to other studies in the literature. (Huang et al., 2000; Sukharamwala et al., 2012; Saraee et al., 2015)

Finally, only 20% of patients were lost during follow up, and this proportion was lower than that found in a study investigating the quality of life after PD (Carter et al., 2009).

## 5. Conclusions

Surgical treatment of periampulary pancreatic cancer with PD according to early significant statistical survey data using the (EORTC) QLQ-C30 version3.0 and the EORTC QOL-PAN26 questionnaires shows that:

o There was no significant change in the general health during follow-up, according to the values of the scale of the QLQ-30 questionnaire
o During follow-up, a significant reduction of pancreatic pain and liver symptoms was found, as was the case with other studies
o The average length of stay in the “Attikon” University General Hospital (Chaidari) where the present study in this sample was carried out, was 11.8 days (SD = 8.3 days), time comparable, to other studies.

This study found the need in our country carrying out large-scale studies to become known and depth evaluation of quality of life of patients after PD.
